# Bioclimate analogue regions - finding present day examples for future bioclimatic conditions

**DOI:** 10.1007/s00484-025-03052-w

**Published:** 2025-10-14

**Authors:** Niels Döscher, Julia Mietz, Alexander Graf, Pablo Fernández de Arróyabe Hernáez, Michael Leuchner

**Affiliations:** 1 Physical Geography and Climatology, Institute of Geography, RWTH Aachen University, Aachen, Germany; 2 Agrosphere Institute, Forschungszentrum Jülich GmbH, IBG-3, Jülich, Germany; 3 Geography and Planning Department, University of Cantabria, Santander, Spain

**Keywords:** Climate analogue regions, Bioclimate, UTCI, CORDEX, Heat stress

## Abstract

This study combines the method of climate analogue regions with a bioclimatic approach. Bioclimate analogue regions were determined for the Rhenish lignite mining area in western Germany, which will face a major structural change in the following decades. These analogue regions currently experience a similar number of days with heat stress compared to the projected future (RCP8.5) at the end of the century in the investigation area. The method is based on the Universal Thermal Climate Index (UTCI) parameters temperature, solar radiation, wind and relative humidity in 3-h temporal resolution while taking day- and night-time values into account. The analogues were calculated for an ensemble of 15 GCM-RCM model combinations from EURO-CORDEX data. The results suggest that analogue regions of the Rhenish lignite mining area are most likely to be found in southern Europe. The highest similarities for the whole ensemble can be found around the Gulf du Lion in southern France. However, some other regions, e.g. around the Black Sea, north of the Balkan Mountains or south of Bordeaux are good fits in some individual model results. While some of these regions are in accordance with previous studies on climate analogue regions, some others were unexpected. The study further shows advantages of using full-coverage instead of punctual data for climate analogue determination, as the results in this study exhibit a high level of spatial detail. For areas facing major structural changes, knowledge of possible climate futures and their present examples can be key aspects for regional planning.

## Introduction

One of the global consequences of climate change that has already been experienced in recent years, is an increasing number of extreme heat events and their impact on human health. For the near future, a further increase of intensity and frequency of possibly dangerous heat-humidity conditions is expected, affecting human morbidity and mortality (IPCC 2023b). There was a large number of studies dealing with this topic in recent years. Ebi et al. (2021) give a general overview of key factors and challenges related to heat stress, whereas other authors address the topic in a regional context, also highlighting social factors of heat vulnerability (Arifwidodo and Chandrasiri 2020; Błażejczyk et al. 2018; Cleland et al. 2023; Pasquini et al. 2020; Winklmayr et al. 2022). The issue is also relevant in the context of increasing global urbanisation (United Nations 2019), because specific climatic effects of cities, particularly the Urban Heat Island (UHI) effect, can amplify heat stress events (Kim and Brown 2021).

Studying the effects of climate, respectively weather, on the human organism is one of the core interests in the research field of biometeorology. To better describe thermal human comfort or discomfort in relation to meteorological parameters like air temperature, humidity or radiation, a large number of biometeorological indices have been developed in the past (Blazejczyk et al. 2012; Freitas and Grigorieva 2015). One of the most widely used is the Universal Thermal Climate Index (UTCI). It was initiated by the International Society of Biometeorology and developed in cooperation with the European Union (Jendritzky et al. 2012). Among other fields of application, it was specially designed for use in climatological studies dealing with heat related health impacts of climate change (Jendritzky et al. 2012). The index was designed as an equivalent temperature to a reference environment, with values given in °C, and is derived from the variables wind speed, radiation, humidity and air temperature (Bröde et al. 2012). As intended, it was recently used in several climatic studies. Cheung and Hart (2014) investigated future thermal comfort in Hong Kong based on UTCI values derived from climate models, while Kjellstrom et al. (2018) focused on future heat exposures of workers by analysing UTCI and Wet Bulb Globe Temperature (WBGT). Brecht et al. (2020) and Cardoso et al. (2023) derived UTCI values from Regional Climate Models (RCMs) to investigate possible future heat stress in Germany respectively Portugal. Beside studies deriving UTCI from climate models, it is regularly used in studies focusing on thermal stress on a local or urban scale (Krüger 2021). There are some challenges in the application of UTCI that need to be considered, especially when applying UTCI to urban environments, such as modelling accurate wind speed in complex urban morphology (Park et al. 2014) or biases from climates models.

One particular way to look at the possible future climate of an area of interest (AOI) and make the related change more understandable for a broader public, is the concept of climate analogues. In the context of climate change, two places are called climate analogues, if the future climate of place A (typically the AOI) is similar to the present climate of place B. The goal is therefore, to identify B as a real-world example of the future climate situation at A. There are several studies in which different methods for the determination of climate analogues are presented. One of the main topics is the calculation of climate analogue cities in order to investigate the effects of climate change on health or economy in urban areas (Bastin et al. 2019; Fitzpatrick and Dunn 2019; Hallegatte et al. 2007; Kopf et al. 2008; Reuter et al. 2023; Rohat et al. 2018). The spatial shift of plant and animal habitats due to climate change and the question if there might be a “no-analogue future” for species is another main interest in climate analogue studies (Parks et al. 2023; Stralberg et al. 2009; Williams and Jackson 2007). Closely related to the latter are studies dealing with future growing areas of crops (Burke et al. 2009; Chaudhary et al. 2023; Pugh et al. 2016).

The key question when determining climate analogues is how to define what similar climate is. Nearly all studies mentioned above give a different answer to this question, usually depending on the thematic focus. Climate parameters like annual mean temperature and sum of precipitation are used in different combinations and with different weighting factors. Sometimes simple indices such as the annual number of hot days are also calculated. This lack of a standardized way to define the climate which is compared between the AOI and possible analogues, makes it hard to compare the results of different analyses. When it comes to human thermal comfort, the beforementioned bioclimatic indices are, in fact, a well proven, standardized way to combine the influence of different climatic parameters. Additionally, they ensure a good comparability between studies, especially when it comes to widely used indices like UTCI. It is therefore a reasonable approach to use them in order to search for bioclimatic analogues. The basic idea was applied by Wang et al. (2022) for a set of 352 Chinese cities, though the UTCI was not directly calculated from climate model data in this study. Instead, future UTCI values were derived by training a random forest algorithm with past UTCI, air temperature and precipitation values. Radiation, wind speed and humidity, which are essential for UTCI calculation (Bröde et al. 2012), were not considered. Additionally, a monthly temporal resolution was used. This might be too coarse given the frequent occurrence of heat stress events with a duration on the order of magnitude of days, several of which might occur in a single month.

Therefore, we propose an alternative way to compute bioclimate analogue regions based on UTCI, with a focus on the occurrence of heat stress situations. In this study a large multi-model ensemble consisting of 15 combinations of global climate models (GCM) and regional climate models (RCM), produced within the framework of the Coordinated Regional Climate Downscaling Experiment (CORDEX) (Giorgi et al. 2009), is used. We consider day- and night-time UTCI values, computed from model outputs of air temperature in 2 m, radiation, humidity and wind speed. For an AOI in western Germany which is mostly characterised by agriculture, small settlements and three large open-pit mines and will face a fundamental structural change in the next decades, we asked the question if there are analogue regions in other parts of Europe, where the annual number of days with heat stress in the recent past is similar to that of the AOI at the end of the century. Unlike many of the studies cited above, which have a focus on climate analogues cities, we did not focus explicitly on urban areas and urban effects but compared an AOI with a complex land-use setting to all possible European regions of the same size. However, the method is technically applicable to any other AOI. Based on previous studies dealing with climate shift and climate analogues, we hypothesise that these locations can be found in several regions across southern Europe. With this study, we are deliberately focussing on one important aspect of human health in the context of climate change and therefore do not claim to show analogue regions of the complete climate. The novelty of the study is the calculation of analogue regions based on UTCI with all relevant parameters from RCM output.

## Materials and methods

This chapter starts with a short overview of the study area and why it was chosen, before continuing with a description of the climate data and how it was processed. Finally, the methodology for the determination of the bioclimate analogue regions is explained.

### Study area

**Fig. 1:**
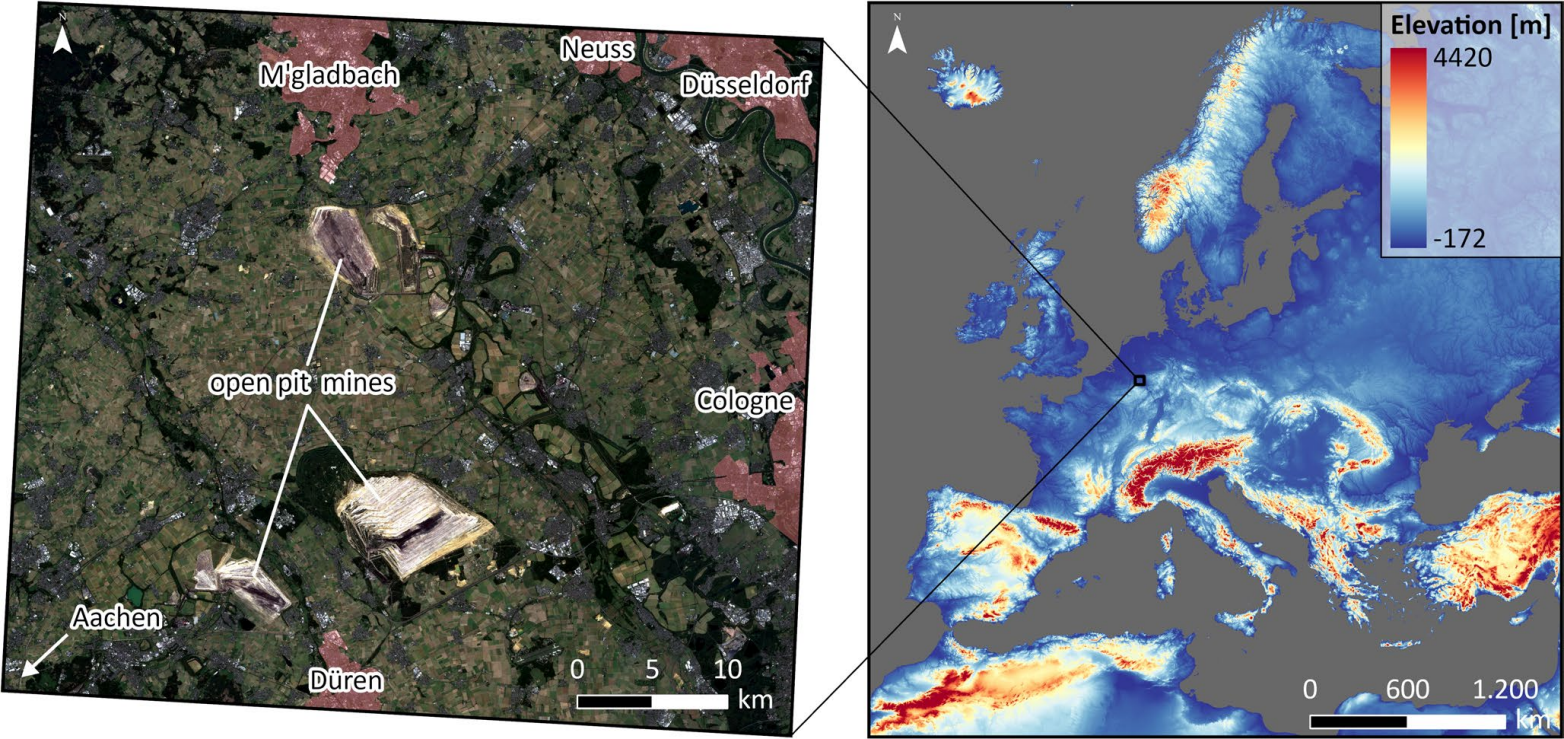
Overview and location of the study area. Left: True colour composite of the study area as seen by ESA’s Sentinel-2 satellite (Drusch et al. 2012). The image is a median of all images of the study area between 2020 and 2023 with a cloud cover of less than 20%. Additionally, the settlement areas of the bordering larger cities (red) and the location of the three large open pit mines are shown. Right: Location of the study area in Europe with the GMTED2010 digital elevation model (EROS Center 2017)

Bioclimatic analogue regions (BAR) were calculated for the Rhenish lignite mining area (RLMA), located in western Germany, between the cities of Cologne, Aachen and Mönchengladbach. Although lignite has been extracted from small pits in this area since the 19th century, three large open-cast mines were established in the 1950s to reach deeper deposits of the resource for electricity generation (Dickmann 2011). The three largest mines Garzweiler, Hambach and Inden currently have a total operating area of about 97 km^2^ (RWE 2021a, 2021b, 2022). However, lignite electricity production phase-out is already legally mandated for the RLMA by 2030 (BfJ 2023, German). As a result, the mines will also be closed at this point, leading to a major structural change in the region in the following decades. Although surrounded by several large and medium-sized cities, the landscape of the RLMA is mostly characterised by agriculture and small settlements (Fig. 1). Because we compare our study area to all different kinds of regions across Europe and due to shortcomings of RCMs regarding urban climate effects, we intentionally chose a non-urban study area with a complex land-use setting.

The planed future use of the mining sites for touristic, recreational or economic uses imposes many challenges, especially regarding the creation of functioning ecosystems which can provide crucial services for human well-being (Leuchner et al. 2024). Their embedding in the still predominant agricultural areas around the mines and the socioeconomic development, also in the adjacent larger cities, is a complex task (Gerwin et al. 2023). The knowledge of climate change can be a key factor in the planning, e.g., for considerations regarding suitable tree species for reforestation or to estimate the need for countermeasures during periods of heat stress. The time horizon of the structural change until the end of the century aligns with that of climate model data. It is therefore possible for this study to provide a fitting time frame for expected change of heat stress situations and to provide examples of other European regions of the same size which presently have similar conditions. A satellite image with land use information of the exact extent of the RLMA as defined in this study as well as the European comparison area is given in Fig. 1.

### Data

RCM data was derived from the European domain of the CORDEX initiative (EURO-CORDEX) (Jacob et al. 2014). The data used here has a spatial resolution of 11° (~ 12.5 km), a temporal resolution of 3 h and covers the European continent as shown in Fig. 1. The data was downloaded from the Earth System Grid Federation (ESGF) node hosted by the German Climate Computing Center (Deutsches Klimarechenzentrum, DKRZ) (https://esgf-data.dkrz.de/search/cordex-dkrz/). RCMs provide climate projections with a relatively high spatial resolution over a limited area, e.g., a continent, and can provide climate data on a regional scale. They are less suitable for analysis on a local, e.g. urban, scale as the spatial resolution is not sufficient. They are driven by GCMs which cover the entire Earth but have a much coarser spatial resolution. A single RCM can be driven by different GCMs. In this study we used a multi-model ensemble consisting of 15 RCM-GCM combinations which are listed in Table 1. The combinations were chosen for two main reasons. Firstly, their simulations include all the necessary variables to calculate the UTCI, which are 2 m surface relative humidity (hurs), surface thermal radiation downward (rlds) and upward (rlus), surface solar radiation downward (rsds) and upward (rsus), 10 m wind speed (sfcWind) and air temperature in 2 m (tas). Secondly, valid wget-scripts for downloading the simulation data were available from the ESGF node. Results for the RCM MOHC-HadREM3-GA7-05 were partly calculated but finally left out, because there were data gaps in the years 2005 and 2099 for some combinations. If there was more than one simulation of the same RCM-GCM combination with different ensemble members or downscaling realisations, we used only one to have the same number of simulations per RCM, in order to avoid biases towards a certain RCM. In the following the simulations will be referred to by their number and letter combination in column N. Each number stands for a single RCM, each letter for a single driving GCM.

**Table 1:** RCM-GCM combinations used to calculate bioclimate analogue regions. For detailed information about the RCM-GCM combinations used in CORDEX see IPCC 2023a

*N*	RCM	GCM	Ensemble Member	Downscaling Realisation
1a	COSMO-crCLIM-v1-1	ICHEC-EC-EARTH	r1i1p1	v1
1b	COSMO-crCLIM-v1-1	CNRM-CERFACS-CNRM-CM5	r1i1p1	v1
1c	COSMO-crCLIM-v1-1	MOHC-HadGEM2-ES	r1i1p1	v1
1d	COSMO-crCLIM-v1-1	NCC-NorESM1-M	r1i1p1	v1
1e	COSMO-crCLIM-v1-1	MPI-M-MPI-ESM-LR	r3i1p1	v1
2a	DMI-HIRHAM5	ICHEC-EC-EARTH	r3i1p1	v2
2b	DMI-HIRHAM5	CNRM-CERFACS-CNRM-CM5	r1i1p1	v2
2c	DMI-HIRHAM5	MOHC-HadGEM2-ES	r1i1p1	v2
2d	DMI-HIRHAM5	NCC-NorESM1-M	r1i1p1	v3
2e	DMI-HIRHAM5	MPI-M-MPI-ESM-LR	r1i1p1	v1
3a	KNMI-RACMO22E	ICHEC-EC-EARTH	r1i1p1	v1
3b	KNMI-RACMO22E	CNRM-CERFACS-CNRM-CM5	r1i1p1	v2
3c	KNMI-RACMO22E	MOHC-HadGEM2-ES	r1i1p1	v2
3d	KNMI-RACMO22E	NCC-NorESM1-M	r1i1p1	v1
3e	KNMI-RACMO22E	MPI-M-MPI-ESM-LR	r1i1p1	v1

The results derived from the RCMs were compared to a dataset of UTCI values derived from ERA5 reanalysis (Di Napoli et al. 2020) for the present climatic situation on the European continent. It was downloaded from the Copernicus Climate Data Store (CDS; 10.24381/CDS.553B7518).

### Pre-processing

Data processing was conducted in Python (version 3.11.6). The aim of pre-processing was to calculate the average number of days per year with heat stress, for both the present and future climate, according to each RCM-GCM combination across Europe. A day with heat stress was defined as a day with maximum day-time UTCI > 26 °C and minimum night-time UTCI > 17.62 °C during the following night. The night-time situation was considered because there is evidence of negative health effects from a lack of night-time cooling (Obradovich et al. 2017; Royé et al. 2021). The day-time threshold is defined as the point when humans start to feel moderate heat stress (Bröde et al. 2012). To our knowledge, there is no established UTCI threshold to define a night with a problematic lack of cooling. Therefore, the night-time threshold was derived from a specific heat wave event in Essen, Germany which was associated with increased mortality (Hoffmann et al. 2008). According to the ERA5 UTCI data described above, the average night-time minimum UTCI during this event was 17.62 °C. Essen is a city located within the Ruhr District, one of the largest urban areas in Europe, about 60 km north-east of the RLMA. Due to the spatial proximity, this value was considered to be a suitable night-time threshold for problematic heat events in the study area. To fully understand this seemingly low value, it should be kept in mind that nighttime outdoor UTCI does not directly interact with vulnerable groups. Instead, its input parameters (e.g. air and outdoor radiant temperature) determine how efficiently indoor UTCI in buildings, overheated during the day, can decrease. This, in turn, defines both, human ability to recover during sleep, and the starting point for further building heating during the next day of the heatwave. As a result of the described selection process, we included only those days into our analysis which have both, heat stress during the day and not enough cooling during the night.

The calculations were conducted separately for the present and future climate in each RCM-GCM combination. The present time period was defined from 1993 to 2022, combining the RCMs historical experiment which covers the years until 2005 with the Relative Concentration Pathway 8.5 experiment (RCP8.5) for the years 2006–2022. The cumulative CO_2_ emissions implied by the RCP8.5 scenario closely agree with historical emissions until 2020 and current and stated policy scenarios until 2050, which makes it the most suitable scenario when analysing the first decades of the 21th century (Schwalm et al. 2020). Although RCP8.5 is a high-emission scenario, the probability of future global emissions tracking these high-emissions is large enough to consider it a realistic pathway (Pedersen et al. 2020; Schwalm et al. 2020). A study by Christensen et al. (2018) suggest that there is a probability greater that 35%, that emission concentrations will exceed those of RCP8.5. Consequently, the RCP8.5 scenario has also been chosen for the future period from 2070 to 2099, to show possible analogues for a pessimistic but still plausible future. The complete time range offered by the RCMs was used while at the same time models which do not cover the year 2100 were not excluded. For 1c the future period is 2070–2098 because the year 2099 was missing in that dataset.

The starting point of the pre-processing for each period from each GCM-RCM combination were 210 data files in NetCDF format, each providing data for one year and one variable in 3-h temporal resolution. The files were first filtered by specific time-steps in Coordinated Universal Time (UTC). The day-time was represented by the three time-steps 9–12, 12–15 and 15–18, night-time by 21−0, 0–3 and 3–6. The long time ranges were chosen to make sure that the maximum day-time and minimum night-time UTCI is found in all time zones existing in the European domain.

The pre-processing was then done in three general steps. To enable the algorithm to be used on a standard computer the data was then processed on raster cell level:

1. For each cell in the European domain, one csv-file was created, containing the values of every variable and every time-step for this cell.
2. Using python’s pickle package (https://docs.python.org/3/library/pickle.html), the csv-files were converted to pkl-files for faster data access.
3. The UTCI value for each time step was calculated using the algorithm of the python package xclim (Bourgault et al. 2023). The xclim-function uses the radiation parameters rlds, rlus, rsds and rsus to calculate the essential mean radiant temperature. The rsds parameter is separated in direct and diffuse solar radiation by deriving the ratio of direct solar radiation from rsds, datetime and the cosine of the solar zenith angle. For a detailed description refer to the xclim documentation (https://xclim.readthedocs.io/en/stable/index.html). For the present and future 30-year periods, the average number of days per year were calculated, where the maximum day-time UTCI and the following minimum night-time UTCI were above the thresholds. These days were considered to be days with heat stress.

The average result of step 3 for the present time period across all models was compared with the ERA5-HEAT dataset (Di Napoli et al. 2020), which provides hourly UTCI values from 1940 to the present. With the exception of some alpine regions, ERA5-HEAT shows much fewer annual days with UTCI values above the thresholds. For most of northern Europe the values are below 1, while the CORDEX dataset shows values between 0 and 15. The difference is greater in warmer regions than in colder regions. In some parts of North Africa the difference is over 100 days. However, the general spatial pattern of the two datasets is similar. Regions with more heat days are found in northern Italy, south-western France, southern Spain and along the Mediterranean coast. Regions with few heat days are located in northern Europe and mountainous regions.

A bias correction of the original model data was not performed, because the determination of the BAR (Sect. 2.5) was realised for each model separately. The present time period of one model is compared to the future period of the same model. The goal of this comparison was not to make a statement about how much the UTCI will increase in the future, but rather measuring similarity between the RLMA in the future period and other European regions in the present. As the statistical bias of a model is expected to be the same in both periods, a comparison of these periods in the same model is valid without bias correction.

### Determination of bioclimate analogue regions for the RLMA

The interim results after pre-processing (Section "Pre-processing") were two grids per GCM-RCM combination, showing the annual number of days with heat stress for the present and future time-period. Based on these two grids, possible BAR for the RLMA were then identified separately for each GCM-RCM combination.

The future heat stress situation in the RLMA was derived by calculating the spatial average within its area on the future grid. This value was then compared to the present situation in regions with the same size across Europe. To do this, a moving window of the same size as the RLMA was applied to the present grid, first calculating the spatial average around each grid cell and then taking the absolute difference to the future RLMA value. The final result is a grid showing the absolute difference in annual number of days with heat stress between the RLMA and regions of the same size across Europe. A value of 0 would mean, that in this region there are presently exactly as many days with heat stress per year as it is expected for the RLMA in the future. Therefore, the lower the value, the more likely the region around the grid cell is a BAR for the respective GCM-RCM combination. To identify those regions considered as BAR across all models, the resulting grids of all GCM-RCM combinations were added.

## Results

### Heat stress climatology across the domain

**Fig. 2:**
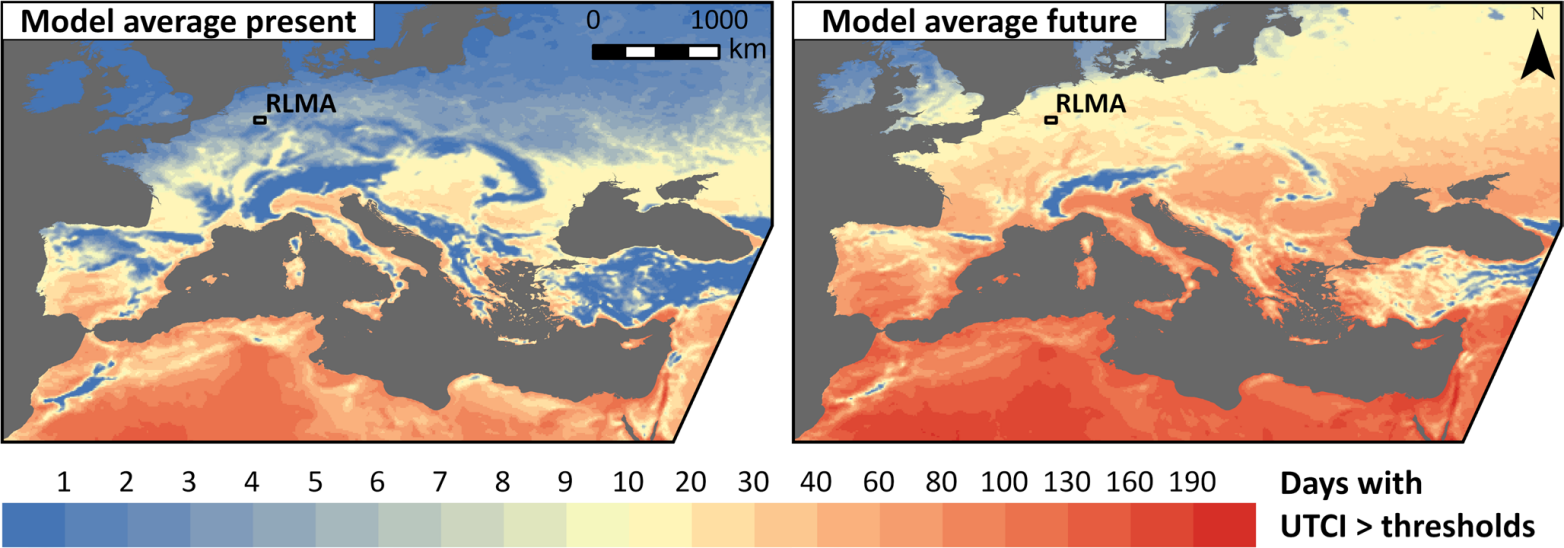
Number of days per year with day-time UTCImax > 26 °C and night-time UTCImin > 17.62 °C for the present (1993–2022) and future period (2070–2099), averaged over all 15 RCM-GCM combinations

Figure 2 shows the ensemble mean of annual number of days per year with heat stress, where the day-time UTCImax was greater than 26 °C and the UTCImin of the following night was greater than 17.62 °C. Similar figures for each RCM-GCM combination can be found in the appendix (Figs. 8, 9, 10 and 11). It is clearly visible that the number of days with heat stress will increase in Europe and northern Africa until the end of the century according to the model data. A closer look at Europe north of the Alps, reveals 0 to 10 days with heat stress in the present period and 10 to 30 days at the end of the century. Only along the northern coasts, in Scandinavia, Great Britain, Ireland and major mountain ranges, values below 10 can still be found in the future period. An exception are some river valleys, namely the upper Rhine, Saone and parts of the Loire catchment, where values above 10 can already be found in the present period. In southern Europe and northern Africa, the values increase as well. While the lower elevation parts of the European Mediterranean coast currently experience about 40 days of heat stress, the model average at the end of the century is between 80 and 130. Looking at the RLMA, the present values range from 4 to 7.5 days in the present and 19.5 to 29 days in the future period. The future RLMA values were used to find bioclimate analogue regions in the present period for each model separately.

### Possible analogue regions for the RLMA according to selected models

**Fig. 3:**
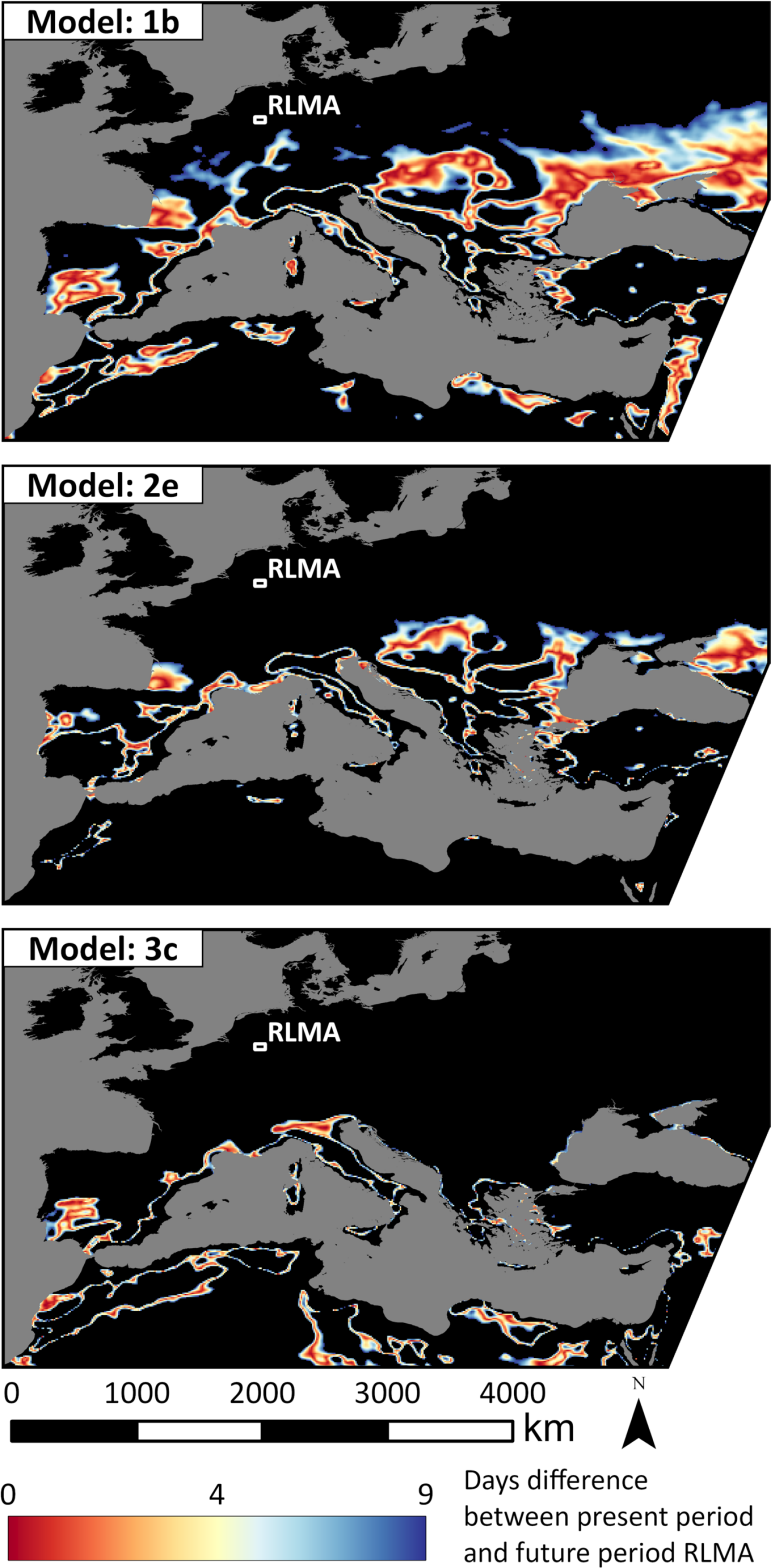
Difference of the average number of days per year with day-time UTCImax > 26 °C and night-time UTCImin > 17.62 °C between RLMA (2070–2099) and other regions across Europe and North Africa (1993–2022), with the same size, for three selected models. Black areas have a difference greater than 9 days

Possible analogue regions for the RLMA according to three selected models are shown in Fig. 3. These models represent the range of results in the ensemble. Similar figures for all models can be found in the appendix (Figs. 6 and 7). The coloured areas show all regions of the present time period, in which the difference in the annual number of heat stress days compared to the future RLMA was 9 or lower which is the 5th percentile of the model average. This ensures that only the most likely analogue regions are displayed in the figures. A value of 0 would mean, that in an area around the cell, which has the same size as the RLMA, there are currently exactly as many heat stress days per year as projected by the model for the RLMA at the end of the century.

In general, a south shift from the RLMA to possible analogue regions can be seen. However, the results of the models show some differences. Model 1b shows a wide spatial range of possible analogues. Major areas with low values are scattered all over southern Europe and northern Africa. They can be found in Spain, France, an area north of the Balkan peninsula, mainly located in Slovenia, Eastern-Austria, Hungary and Slovakia, a large area north of the Black Sea as well as many locations along the Mediterranean Sea. Even the upper Rhine and Saone valleys are visible with values as low as 2.5 days difference.

The regions according to model 2e are fewer and more concentrated than these of 1b. Similarities can be seen in the south of France, the areas around Hungary and some parts along the coast of the Black Sea and the European Mediterranean. However, only very few areas can be seen in northern Africa and the region in south-west Spain is also very different compared to model 1b.

The result for model 3c shows fewer regions than the other models. A main difference is the Po valley in northern Italy, which is a main analogue region in 3c. The region in south-west France is missing here, the region in south-west Spain is visible, if less large than in model 1b. 3c is also the model with the largest southwards shift from the investigation area. Possible analogue regions can be seen even in many areas of northern Africa. In eastern Europe, 3c shows almost no possible analogues.

An interesting feature in all of these maps, are linear areas of possible analogues which seem to follow a certain altitude level in the major mountain ranges. This can be seen in the Southern Alps and Carpathians in 1b and 2e, the Atlas Mountains in 1b and 3c, and the Apennines in all models.

### Possible analogue regions for the RLMA according to the ensemble average

**Fig. 4:**
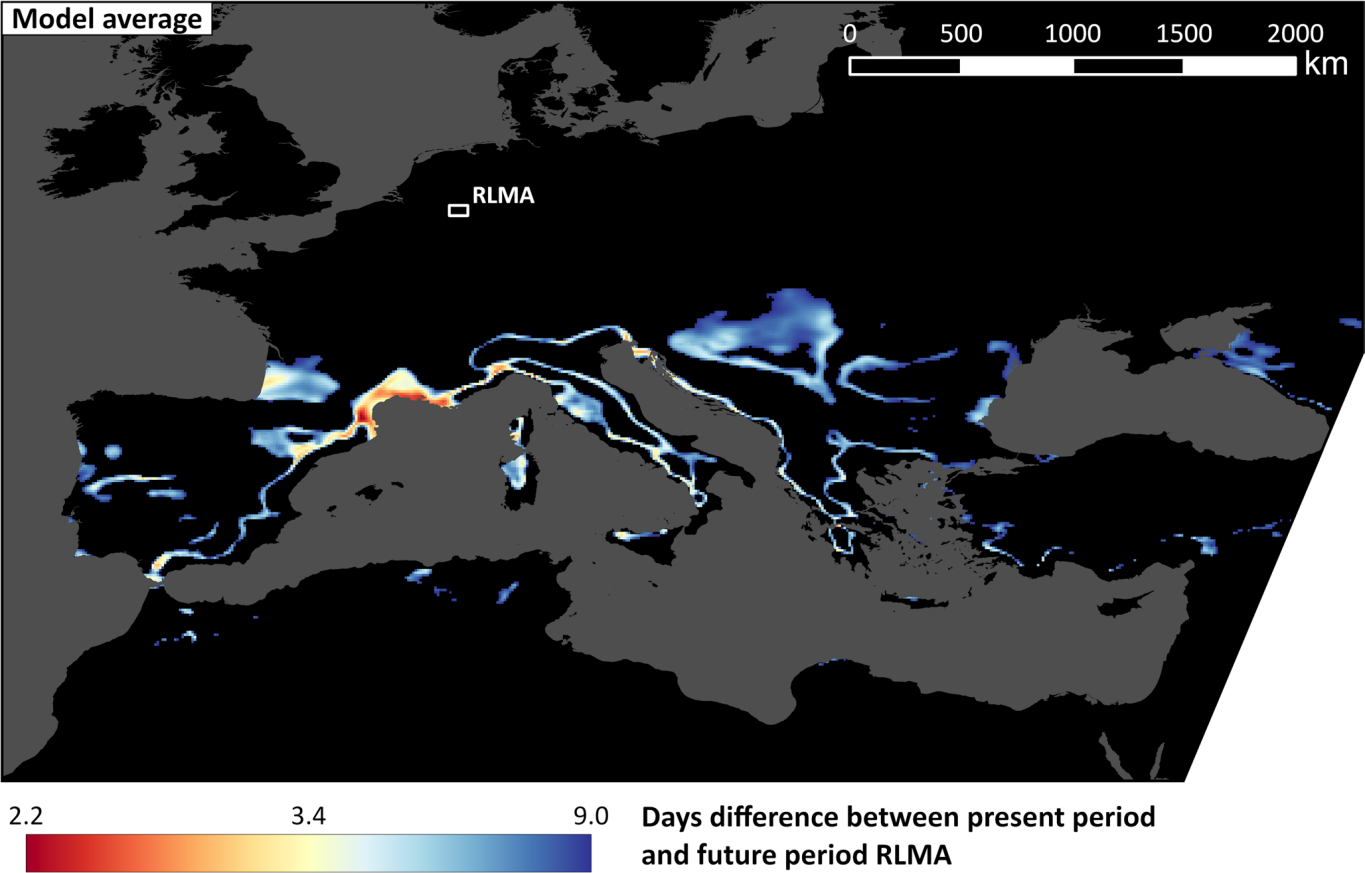
Bioclimate analogue regions of the RLMA. Difference of the average number of days per year with day-time UTCImax > 26 °C and night-time UTCImin > 17.62 °C between RLMA (2070–2099) and other regions across Europe and North Africa (1993–2022) averaged over all 15 RCM-GCM combinations. Black areas have a difference greater than 9 days

Figure 4 summarises the results of the entire model ensemble. It shows the mean of the individual model results, so the coloured areas are on average within the 5% best-fitting analogue regions. The most likely analogue regions are along the coast of the Gulf du Lion in France with a minimum average difference of 2.2 days with heat stress compared to the future RLMA. The values are particularly low in an area east of the city of Perpignan. Other notable areas with high similarity are located in Catalonia, southern Piedmont, Liguria and Istria. Large possible analogue areas, but with less similarity to the RLMA, can be found in southwest France and northwest Spain, Tuscany, Sardinia and north of the Balkan Peninsula. Also, the linear “mountain analogues” are visible in the southern alps, the Apennines or the Balkan. Generally, possible climate analogue regions are located south of the RLMA.

**Fig. 5:**
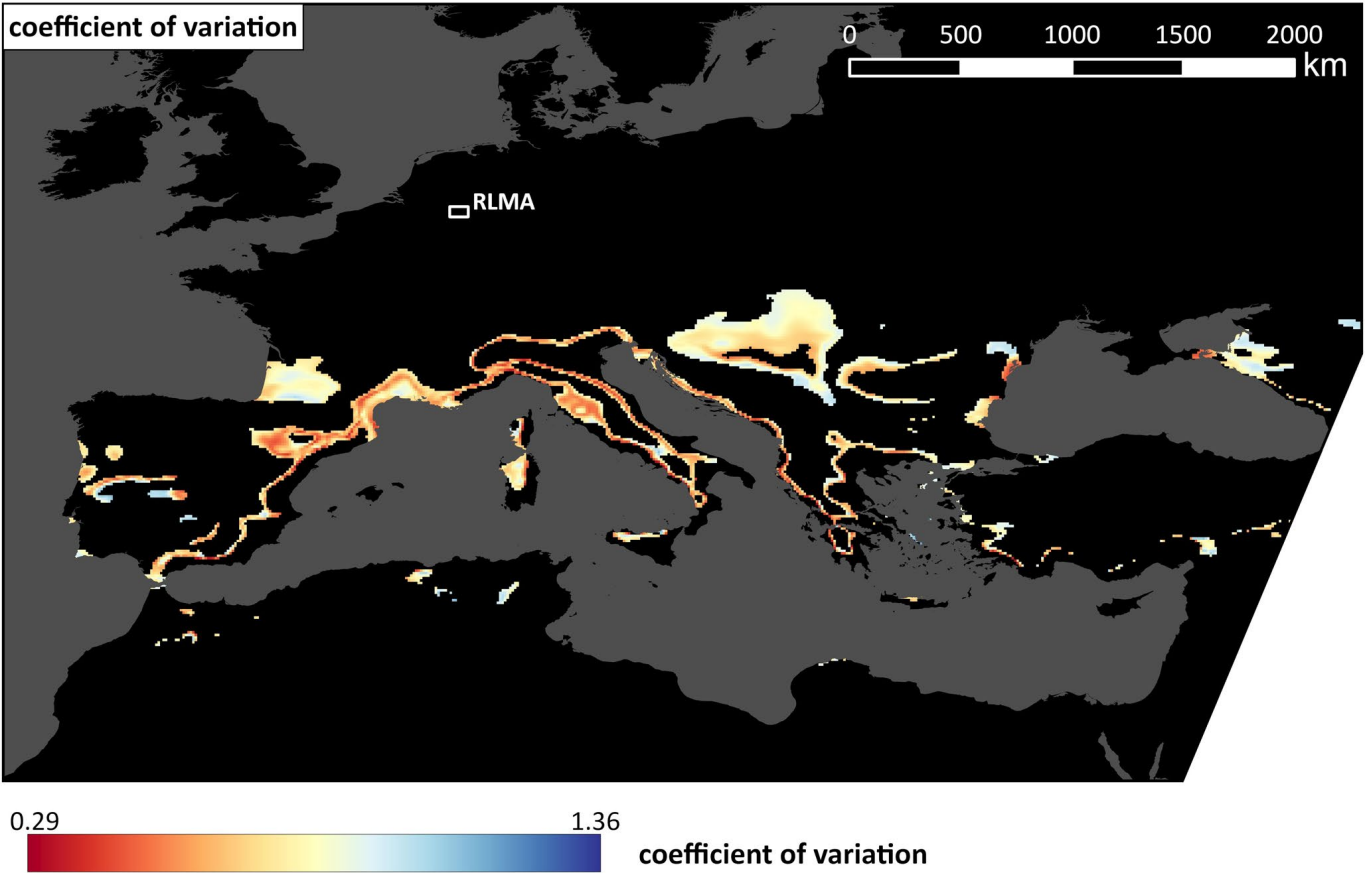
Coefficient of variation, computed by dividing the standard deviation across all 15 RCM-GCM combinations with the model average shown in Fig. 4 for the best fitting analogue regions

To support the findings derived from Fig. 4, Fig. 5 shows the coefficient of variation (CV) as an uncertainty measure for the most likely analogue regions. It is computed by dividing the ensemble standard deviation with the ensemble average (Fig. 4). Areas with low CV are those analogues regions which are most likely to be found across all RCM-GCM combinations. They can for example be found in Spain, Italy, along the Mediterranean coast, at the Black Sea and especially along the linear altitude regions mentioned above. Areas which are among the most likely analogue regions in Fig. 4 and additionally show low uncertainty values in Fig. 5 can be seen around Liguria and some parts of the region at the Gulf du Lion. Some areas along the eastern part of the Gulf du Lion on the other hand show high similarity to the RLMA in Fig. 4, but low CV.

## Discussion

A general expectation of climate change is an increase in temperature and heat stress events (IPCC 2023b). The overall results of this study seem to be consistent with these expectations. All models analysed predict an increase in the number of days with heat stress in Europe based on the UTCI. Accordingly, possible bioclimate analogue regions of the RLMA can be found in southern regions of the investigation area that tend to be warmer.

### Comparison with other studies

In this study an AOI with diverse land-use was compared to all possible regions of the same size in Europe. Although many previous studies regarding European climate analogues were limited to urban areas and analysed the overall climate instead of focusing on heat stress, the results seem to show some convergence. A general southward shift from the location of cities to their respective climate analogues was also investigated by Rohat et al. (2018). Bastin et al. (2019), who included major global cities in their study, suggested San Marino and Milan as European climate analogue cities of Cologne, which is located directly east of the RLMA. The region around San Marino lies within the range of possible climate analogues defined in Fig. 4, with values between 6 and 8.5, and is thus considered a good fit by most models in this study. In contrast, the Milan-area is not considered a likely heat stress analogue by the ensemble. It currently experiences more days with heat stress than the future RLMA. However, it is seen as a good fit by models 3a to 3e. Rohat et al. (2017) suggested Bordeaux as a climate analogue city of Cologne for the scenario SRES A2, which can be compared to RCP8.5. Although no clear climate analogue city was found for Aachen using RCP8.5 by Reuter et al. (2023), the city triangle Bordeaux-Toulouse-Bilbao as well as Florene and Prato were among the best fits. This is supported by our findings to a certain extent. The regions around Bordeaux in south-west France and Florence and Prato in Tuscany are among the possible analogues in Fig. 4 and thus show a high similarity to the future RLMA in the model average. In the case of Bordeaux, the highest similarity was not found around the city itself but further south, in an area between Bordeaux and Dax. In Tuscany, Prato is included in the area of high similarity which mainly stretches south-east of the two cities with maximum values north-east of Grosseto and between Livorno and Piombino. Although the aim of the above-mentioned studies was to identify analogue cities, and therefore only urban areas were included, this finding highlights an advantage of using full-coverage data rather than punctual data, as the results in this study show a higher level of spatial detail. The areas along the Gulf du Lion, the most likely analogues according to the ensemble of this study, have not been mentioned as a climate analogue for western Germany in previous studies to our knowledge. As we searched for bioclimate analogues, differences to previous studies could be expected.

### Interpretation of the results

When interpreting the results in Fig. 4 it is helpful to include uncertainty values like the CV in Fig. 5 in order to assess the robustness of the results within the model ensemble. Areas with high similarity to the future RLMA in Fig. 4 and low uncertainty values in Fig. 5 might be seen as the most likely analogue regions across all models. Those areas include regions in and around Liguria in Italy and some parts of the area at the Gulf du Lion. Some other regions at the Gulf du Lion have a higher coefficient of variation. However, they can still be seen as likely analogues as a low average value can lead to a relatively high coefficients of variation, although the sum of standard deviation and average would still be in the value range shown in Fig. 4. Other parts, for example at the black sea, have a relatively high average values but show a high agreement across the model ensemble.

**Fig. 6:**
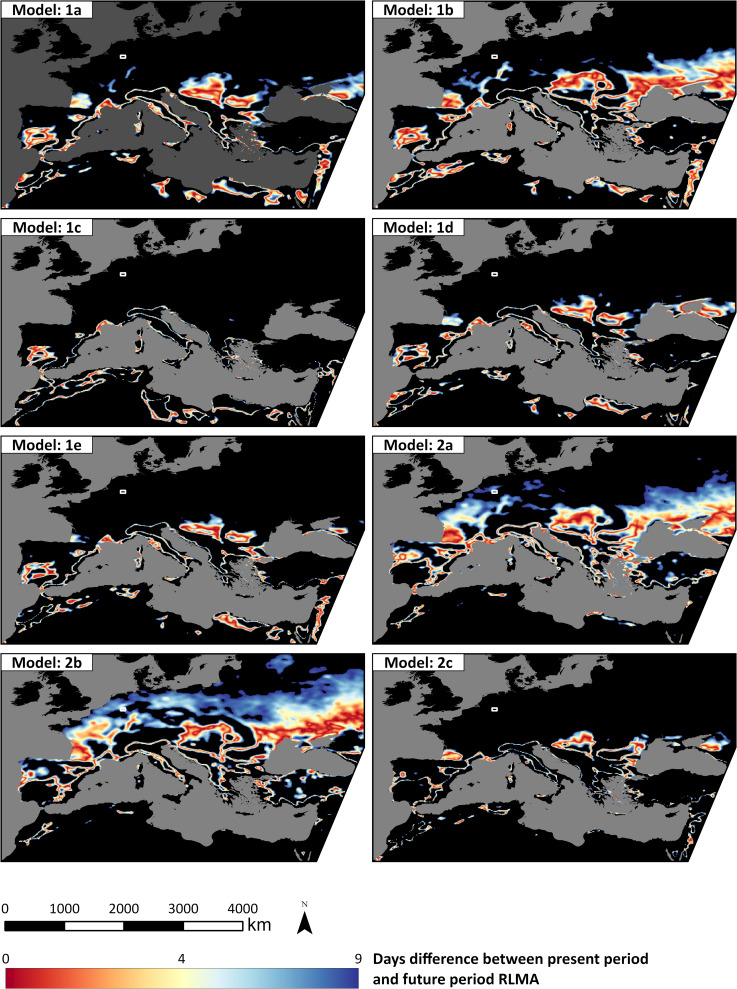
Difference of the average number of days per year with day-time UTCImax >26 °C and night-time UTCImin >17.62 °C between RLMA (2070-2099) and other regions across Europe and North Africa (1993-2022), with the same size, for models 1a to 2c. Black areas have a difference greater than 9 days

**Fig. 7:**
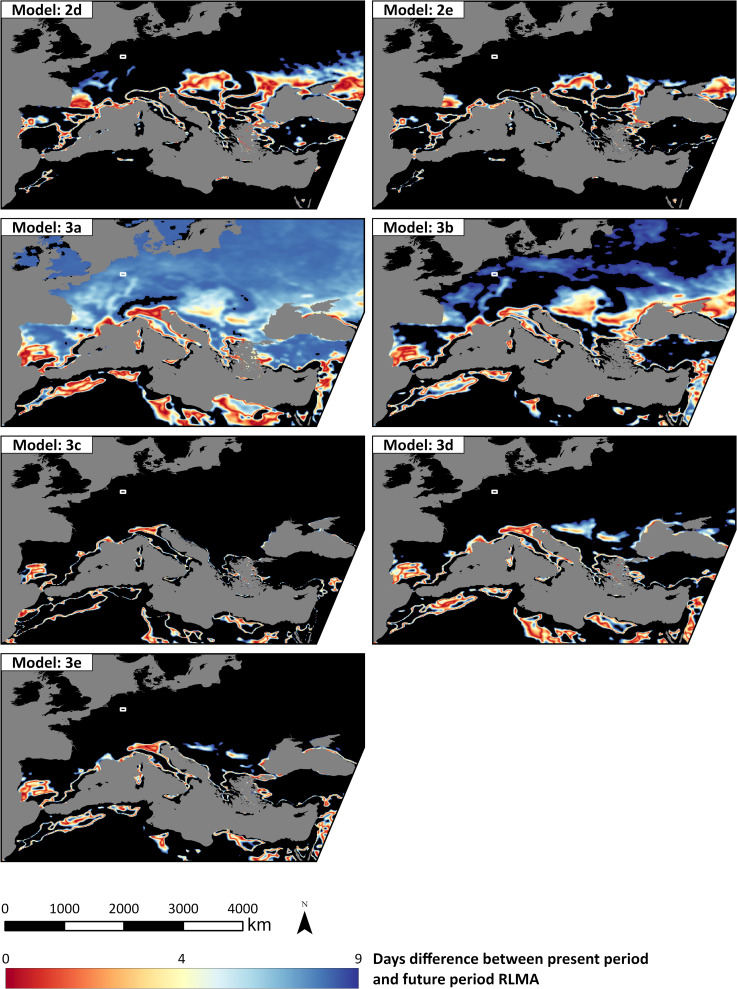
Difference of the average number of days per year with day-time UTCImax >26 °C and night-time UTCImin >17.62 °C between RLMA (2070-2099) and other regions across Europe and North Africa (1993-2022), with the same size, for models 2d to 3e. Black areas have a difference greater than 9 days

**Fig. 8:**
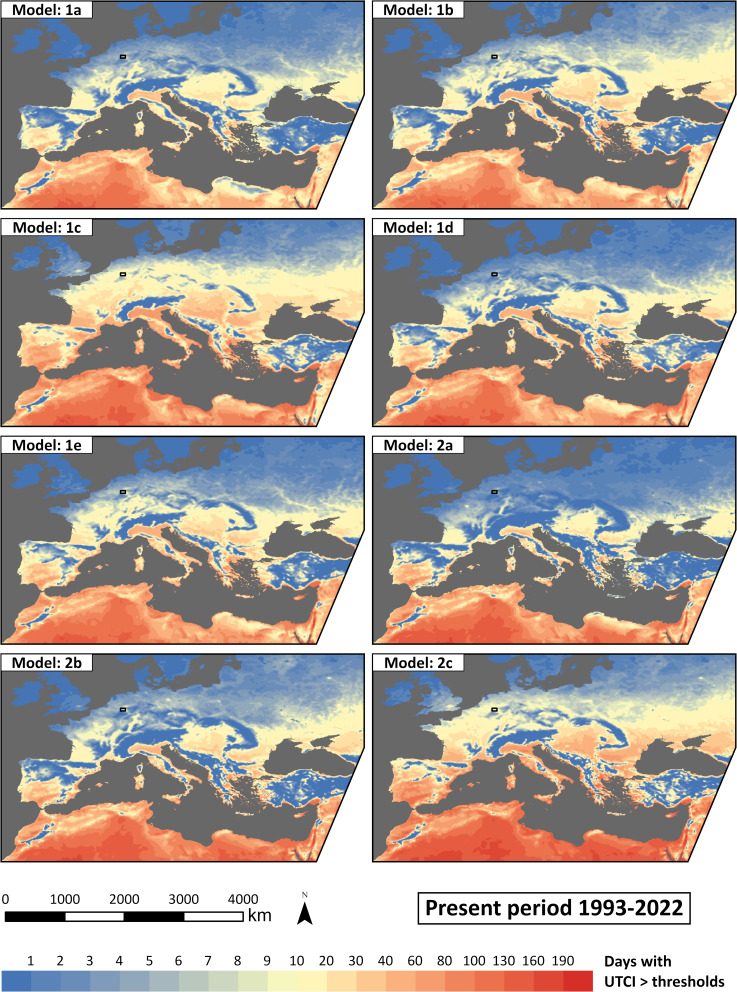
Number of days per year with day-time UTCImax >26 °C and night-time UTCImin >17.62 °C for the present period (1993-2022), for models 1a to 2c

**Fig. 9:**
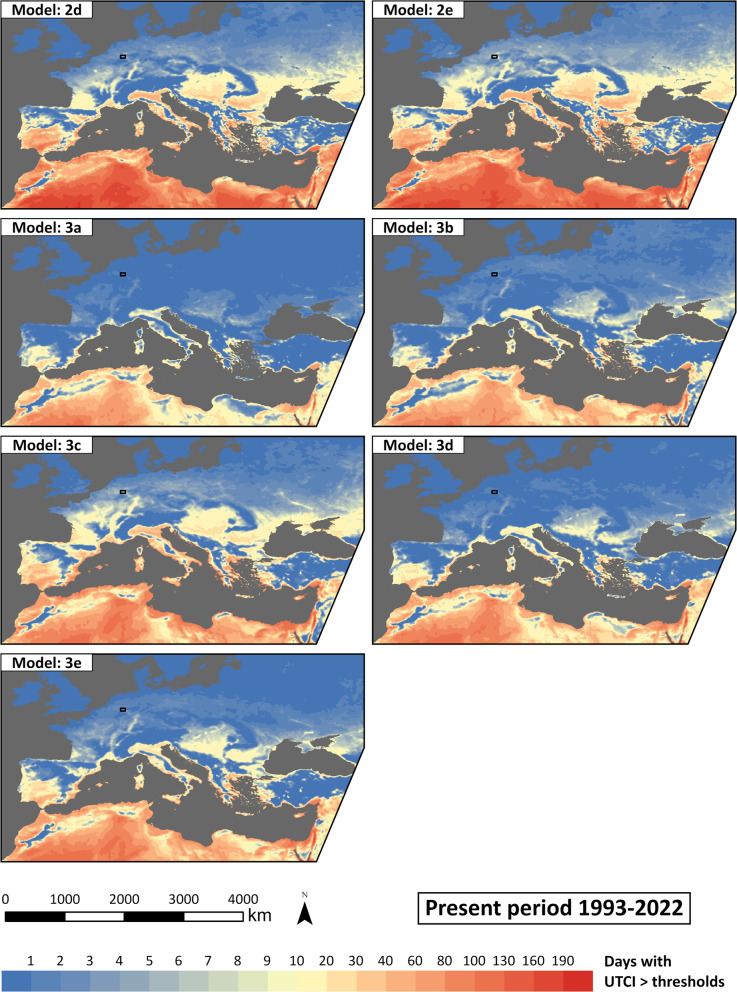
Number of days per year with day-time UTCImax >26 °C and night-time UTCImin >17.62 °C for the present period (1993-2022), for models 2d to 3e

**Fig. 10:**
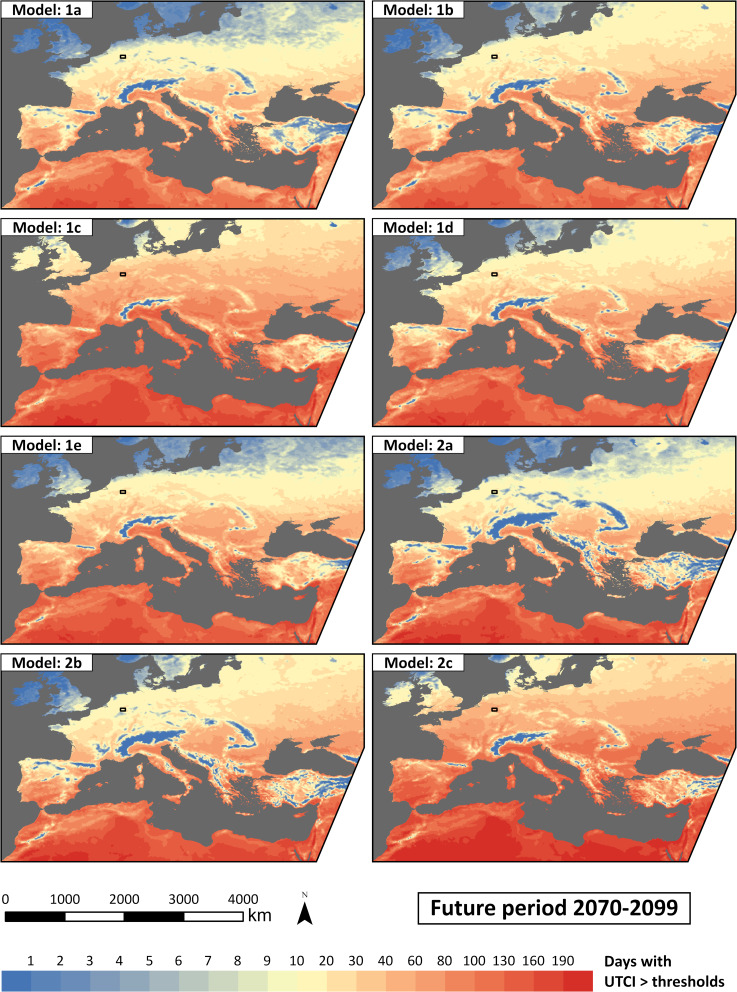
Number of days per year with day-time UTCImax >26 °C and night-time UTCImin >17.62 °C for the future period (2070-2099), for models 1a to 2c

**Fig. 11:**
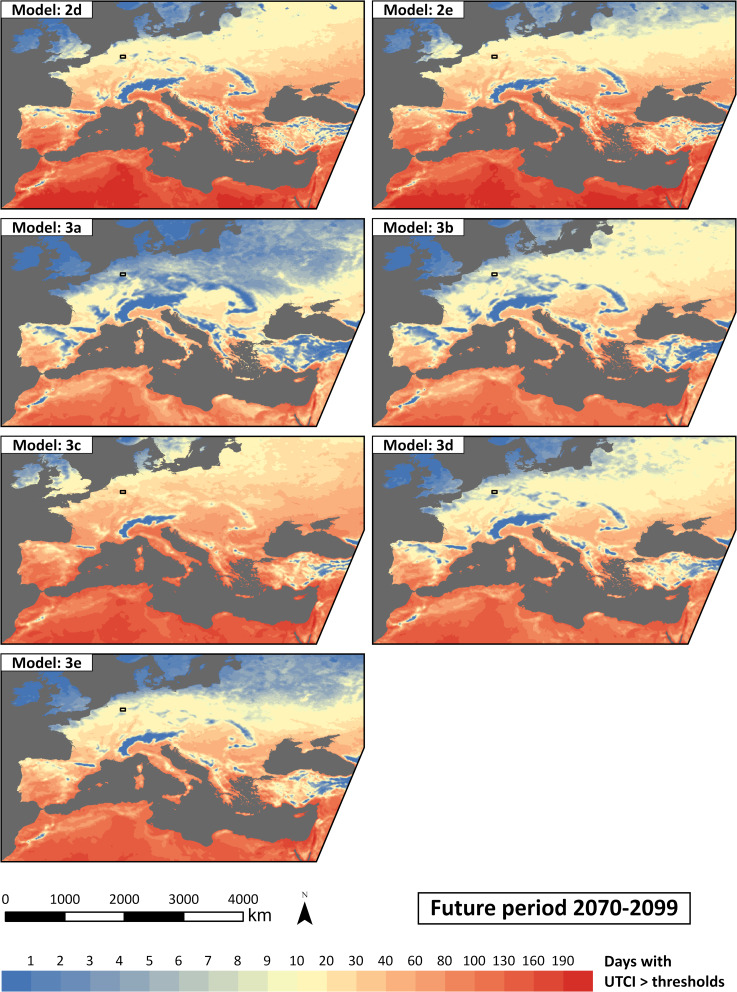
Number of days per year with day-time UTCImax >26 °C and night-time UTCImin >17.62 °C for the future period (2070-2099), for models 2d to 3e

Differences within the ensemble can also be seen in when comparing individual model results (Figs. 6 and 7). In model 3a, for example, large parts of the European continent, including areas north of the RLMA, are within the similarity range of 0 and 9 heat stress days compared to the future RLMA. This is due to very low and homogeneous values in the present period (Fig. 9), which only exceed 10 in a few areas around the Mediterranean. Models driven by the MOHC-HadGEM2-ES GCM (1c, 2c, 3c) on the other hand show very few but well-fitting possible analogue regions. The present period in these models tends to have higher values with a wider range. Because of these differences, it seems reasonable to calculate the analogue regions for each model separately, in order to capture the full possible range of climate analogue regions. For example, almost every model shows well-fitting analogue regions somewhere around the Black Sea, but they are often not in the exact same place, which leads to an underrepresentation of this region in Fig. 4. From the individual model results (Figs. 6 and 7), however, this area is an important candidate for heat stress analogues of the RLMA. The best representation of such results in a synthesis figure or dataset summarizing all models depends on the goal of the synthesis exercise. A geodataset assigning high scores only to exact locations where all or many models agree, e.g. by showing the maximum or a high percentile of the days difference within the ensemble, would be best to pinpoint few locations with a high certainty of being a climate analogue. The opposite (minimum or low percentile of days difference) would reveal the most complete choice of potential candidate regions. Obviously, our Fig. 4 based on arithmetic averaging is a compromise between these two extremes.

It is well known that altitudinal climate zones in mountainous areas can be similar to those in lower regions at other latitudes. For example, the Köppen-Geiger Dfc class describes the climate of the boreal forests of northern Europe, but can also be found at certain altitudes in the Alps (Kottek et al. 2006). The results show that this concept also applies for climate analogue regions. On the maps, prominent linear patterns can be seen, apparently formed along certain elevations of the Alpes, Apennines or the Balkan Mountains. Above these zones the number of heat stress days are too low and below them too high to be comparable with the future RLMA. Although, the terrain of these “mountain analogues” is too different from that of the RLMA to consider them as good candidates for analogue regions, it is an interesting visualisation of the interaction between climate and altitude.

### Limitations of the presented method

The method presented here still has some limitations and shortcoming which must be kept in mind when interpreting the results. RCMs are, for example, not able to show urban climate effects due to their limited spatial resolution and lack of urban canopy models. To adequately include effects like UHI, CORDEX data for the city locations would need to be downscaled while including urban climate models into the analysis (Le Roy et al. 2021). Urban specific biases might also include an underestimation of UTCI compared to the shown results. Similar to the “mountain” analogues this could result in a pattern where “city analogues” are spatially distributed at specific points, within surrounding areas that have less days with heat stress. Even though RCMs cannot show intra-urban climate, they might show the regional climate in which a city finds itself. We would argue that urban climate follows comparable rules in every city, with stronger effects in larger and more densely build-up urban areas. The respective UHI effect would then amplify the climate change effects of the city’s region.

An important aspect when analysing negative effects of heat stress are differences in vulnerability and adaptive capacity which might occur between the RLMA and other regions in Europe or within regions itself. The same heat stress values might have a different effect on different groups of people considering their health and age, ability to protect themselves from the heat or access to health care (Navas-Martín et al. 2024). Despite that, UTCI as a well-known and widely used index, was connected to health data in various studies (Błażejczyk et al. 2018; Nastos and Matzarakis 2012; Romaszko et al. 2022), This allows the results to be compared with those of investigations in the determined analogue regions, which have associated UTCI with mortality (Di Napoli et al. 2018).

Another limitation is the night-time threshold used in this study. It was derived from a single heat event near the RLMA with a proven effect on cause-specific mortality. For the robustness of our method, it would be of great advantage to include a robust critical night-time threshold based on multiple heat events with an in-depth validation against mortality data. Such an analysis was not achievable during this study. However, we would welcome future research in this area.

Additionally, the climate model data does not account for possible land use changes in the RLMA, which may alter the input parameters of the UTCI. However, it is difficult to adequately predict future land-use changes, which would add an additional uncertainty to the method while knowledge of the of climate change effects for the current land-use setting might be valuable for planning the future land-use development of the area in one or the other direction. Particularly for areas undergoing major structural change in the coming decades, such as the RLMA, knowledge of climate change and its impacts in the region could have a positive impact on planning (Becker et al. 2022). Knowledge of the potential challenges to human health under new heat stress conditions, and of real-world examples that could serve as models for dealing with these conditions, can contribute to these positive impacts.

## Conclusion

Visualisation of climate change is a key aspect of the climate analogue region approach. Comparing the future climate of a place with a current real-world example, which one might know from personal experience, makes it easier to imagine what future conditions might feel like and what challenges might come with it. The approach of this study focuses on those conditions, which are actually challenging for human health. The well-known UTCI is used to determine possible bioclimate analogue regions for the RLMA in Europe and parts of northern Africa, based on the number of annual heat stress days. It is shown that reasonable results can be obtained when determining climate analogue regions with the help of bioclimatic indices.

The analogue regions are mainly located in southern Europe and northern Africa, indicating an expected increase of heat stress in the RLMA. While different RCM-GCM combinations show different analogue patterns, there are areas with high similarity throughout the entire model ensemble which can be interpreted as the most likely analogues regions.

While we present analogue regions with respect to the RLMA, our methodology, data source and code could be re-used for other AOIs. Without changing the raw data source, such a AOI should not be near an edge of the domain, and for projections into a warmer future in the northern rather than the southern half of the domain. In the corresponding analogue plot of Fig. 4, resulting from such an analysis, a decrease of the difference at the southern edge of the domain (as opposed to the local minima) would indicate that the domain was inadequate for the AOI and question. Especially for regions which face major changes in the future, might profit from knowledge of their climate analogues from a planning perspective.

## Data Availability

The CORDEX regional climate model data, used for the determination of the bioclimate analogue regions, is available at the Earth System Grid Federation (ESGF) node hosted by the German Climate Computing Center (Deutsches Klimarechenzentrum, DKRZ), https://esgf-metagrid.cloud.dkrz.de/search/cordex-dkrz/. UTCI values derived from ERA5 reanalysis data, which was compared to the CORDEX Data for the present period, is available from the Copernicus Climate Data Store (CDS), 10.24381/CDS.553B7518. The datasets generated during the current study are available from the corresponding author on reasonable request.
